# Fertility-Enhancing Potential of *P. amygdalas* and *J. regia* Oil Mixture in Wistar Rats: Male/Female Infertility Models Assessment

**DOI:** 10.1155/sci5/3936227

**Published:** 2025-04-29

**Authors:** Sadia Suri Kashif, Sadaf Naeem, Saira Saeed Khan, Shaheen Perveen, Nausheen Alam, Saba Zubair, Javeria Ameer

**Affiliations:** ^1^Faculty of Pharmacy and Pharmaceutical Sciences, Ziauddin University, Karachi, Pakistan; ^2^Faculty of Pharmacy and Pharmaceutical Sciences, University of Karachi, Karachi, Pakistan; ^3^Institute of Pharmaceutical Sciences, Jinnah Sindh Medical University, Karachi, Pakistan; ^4^Faculty of Pharmacy, Federal Urdu University, Karachi, Pakistan

**Keywords:** almond, fertility, mixture, oxidative stress, sex hormones, two-generation study, walnut

## Abstract

Polyunsaturated fatty acids–rich nuts are a group of natural sources that have served as a tonic in the treatment of many ailments for centuries. In this regard, *P. amygdalas* and *J. regia* nuts are traditionally used in infertility treatments. However, so far, the main mechanisms for the fertility-enhancing effects of these nuts in males/females are unknown. The present study was intended to evaluate the fertility-boosting effect of a mixture constituting *P. amygdalas* and *J. regia* oil on male/female infertility models and in two successive generations of rats; *F*_0_ (parents) and *F*_1_ (offspring). For the fertility assessment, male and female rats, 18 pairs (*n* = 36, 1:1, *F*_0_ generation), were separated into three groups and dosed with 2 and 4 mL/kg daily of oil mixture and saline, respectively, from precohabitation up to lactation. To determine the further protective role of the oil mixture in infertility, we designed ethanol-induced male and estradiol-induced female infertility models. Various parameters like hormonal, hematological, lipid profile, and antioxidant markers [superoxide dismutase (SOD), glutathione peroxidase (GPx)] were measured along with histopathology of sex organs. The continual exposure of *F*_0_ and *F*_1_ generations to the oil mixture did not affect the fertility index and survival index in females. However, in males, all sperm parameters were significantly improved in both generations. We have found pronounced fertility outcomes with oil mixture supplementation in both genders of *F*_0_ and *F*_1_ generations. Results showed that the oil mixture significantly restored (*p* < 0.05) luteinizing hormone (LH), follicular stimulating hormone, GPx, plasma testosterone, and SOD activities in both models. Histological findings endorsed enhanced folliculogenesis and spermatogenesis with enhanced architecture. Our results strongly suggest that *P. amygdalas* and *J. regia* oil mixture might be a promising option in future for male/female sterility treatment. This finding could pave the way in infertile men and women for clinical trials. This is the first study that has provided an experimental rationale for a walnut and almond oil mixture combination as an effective candidate for fertility recovery by improving sex hormones and managing oxidative stress.

## 1. Introduction

Infertility is referred to as couple's difficulty in conceiving after 1 year of marriage. Major causes of infertility are polycystic ovary syndrome (PCOS) and endometriosis in females and low sperm count and testosterone levels in males [1]. In general, 50% of the infertility cases are due to the female factors especially PCOS. PCOS is a common endocrine disorder of fertile females that affects 6%–10% young females worldwide [2]. Frequent symptoms of PCOS are prolonged or irregular menstruation and hyperandrogenism that are associated with oxidative stress, which disrupts ovarian function and hormonal balance [3, 4]. Oxidative stress produces disparity in the generation of reactive oxygen species that generate inflammation and enhances insulin resistance, extracellular abnormal ovarian remodeling, multiple cyst production, anovulation, finally leading to infertility [5, 6]. Similarly in males oxidative stress may alter redox balance in seminal fluid, sperm motility leading to male infertility [7]. The etiology of infertility in male/female is multifactorial, and the accessible treatments have indefinite success rates [8]. However, no specific treatments for improving fertility potential are available. Only assisted reproductive techniques are potentially helpful for infertile couples including, in vitro fertilization, and intrauterine insemination to contribute pregnancy [9].

The lack of available infertility therapies has led to the extensive use of alternative herbal and hormonal therapies. Due to the harmful effects of available chemical drugs on the reproductive health, modern fertility treatment procedures and the high costs of drugs, the inclination to use herbal medicines is enhancing among men and women [10]. The rationale for using these treatments is based on the speculation that oxidative stress and a deficiency in a hormone or vitamin may lead to some kinds of infertility. Alternative therapies (herbal medicines, hormones and vitamins) can improve male/female fertility potential and the quality of semen/ova [9].

Several studies have revealed the role of alternative medicines for treating female/male infertility alone and in combination with other therapies [11]. These include herbal extracts, antioxidants, multivitamins, and fatty acids [linoleic acid, monounsaturated fatty acids (MUFS), polyunsaturated fatty acids (PUFAs), omega‐3, omega‐6, docosapentaenoic acid, eicosapentaenoic acid] [12]. PUFAs containing plants have been consumed for several years in alternative medicine. PUFAs are conceived vital for reproductive health as they can affect fertility in both males and females by manifesting alteration in membrane phospholipids composition during reproductive processes. In females, PUFAs are important in regulating the menstrual cycle, reducing insulin and testosterone levels as well as in restoring metabolic health [13]. They are also involved during the early reproductive phase, including oocyte quality, maturation, and embryo implantation [14, 15]. There has been a growing interest regarding the effects of dietary fatty acid intake on the prevalence of infertility in females [15]. Previous researchers have shown that in males, during sperm maturation, docosahexaenoic acid (DHA) accumulated in the sperm membrane, and was associated with normal morphology, better sperm concentration, and motility. DHA in sperm membrane can also affect crucial fertilization events including acrosome reaction, capacitation, and sperm–oocyte fusion [16, 17]. PUFAs present in various plant seeds like walnut, almond, peanut, etc. are responsible for decreasing oxidative stress. Tree nuts (hazelnuts, Brazil nuts, almonds, walnuts) include vital fertility-boosting minerals that are a rich source of plant-based proteins [18]. In ancient times, crude nuts were consumed by individuals attempting to get pregnant, as they are a fantastic plant source of proteins and PUFAs. Nuts rich in omega-3 fats taken during pregnancy strongly affect a child's behavior [19] as nuts are enriched with micronutrients, macronutrients, and numerous bioactive health-promoting compounds that can regulate male/female fertility parameters [20]. Pakistan is an underdeveloped country with a lack of resources. The inaccessibility of essential equipment and trained medical personnel, along with unaffordability of treatment, are the additional fertility care impediments [21]. As a result, people in Pakistan consume medicinal plants to address their challenges of infertility. Plants employed in Pakistan, for the treatment of male/female infertility include *Mentha piperita, Brassica oleracea, Acorus calamus* [22], *Tamarix aphyla, Melia azedarach* [23], *Tribulus terrestris* [24], etc.

*Prunus amygdalas* L. (almond) belongs to the family Rosacea. It is a significant source of Vitamins and minerals; A, E, D, B_1_, B_6_, magnesium, calcium, zinc, beta-zoosterol, alpha-tocopherol, squalene and was found to enhance levels of superoxide dismutase (SOD) and glutathione peroxidase (GPx), which suggests its potential antioxidant effect [25, 26]. *P. amygdalas* and other nuts are associated with properties that could help treat infertility. Kernels of many kinds of almonds have been proven nutritious for a fertility diet [18]. It was found that zinc in nuts helped eliminate infertility in men [27]. *P. amygdalas* kernels are one of the healthy nuts used for both male and female infertility. They are rich in micronutrients that are believed to help in sperm production. Furthermore, they are the sources of natural antioxidants that assist in spermatozoa production necessary for male sexual hormone regulation [28, 29]. These nuts were also considered superfoods because of their antioxidant properties and capacity for enhancing reproduction [30].

Similarly, *Juglans regia* L. (walnut) also reported for its fertility-boosting effects. It belongs to the family Juglandaceae and contains various bioactive compounds including melatonin and serotonin, rich in magnesium as well as other minerals, and PUFAs [31]. Adelakun et al. studied the procreative abilities of *J. regia* by experimenting on rats and suggested that male rats displayed enhanced reproductive capacity after taking *J. regia* oil by enhancing sperm count [32]. Ikwuka et al. also endorsed this finding that the consumption of *J. regia* frequently boosts male fertility in a study on Wistar rats. This sperm count and motility improvement might be owing to the presence of glycosides, alkaloids, and saponins, which possess antioxidant potential and are known to inhibit malondialdehyde (lipid peroxidation end product). Moreover, it was also reported earlier that saponins may improve sperm parameters [33]. Similarly, Masterson et al. in their study found *J. regia* consumption improves the semen quality, sperm count, and fertility [34].

Nuts are enriched with PUFAs that have diverse biological effects on insulin sensitivity, insulin levels and insulin secretion. Insulin resistance reduces SHBG (sex hormone–binding globulin), that reduces testosterone binding and consequently enhances free testosterone. Nuts including almonds and walnuts have a favorable effect on testosterone by increasing SHBG, and reducing testosterone [35]. In another study, supplementation with PUFAs improves Testosterone, SHGB and luteinizing hormone (LH) levels in PCOS women [36].

Various researches have been conducted to explore the individual fertility-boosting effect of almond oil [28, 37] and walnut oil [38, 39], but no work has been reported on their combination. The combination of different plants having various phytochemical agents exhibits synergistic and additive antioxidant properties that exhibit health-promoting effects. Thus, the novelty and aim of our work is to explore the fertility augmenting effect of *P. amygdalas* and *J. regia* oil mixture on reproductive parameters and on female and male infertility models.

## 2. Methods

### 2.1. Ethical Approval

The animal experiment was conducted in accordance with departmental Ethics Committee for Animal Care and Use (ECACU) Pakistan. This study was approved by the Animal Ethics Committee (Board for Advanced Studies and Research; reference No 05071/pharm), dated 28 March 2019 [40].

### 2.2. Chemicals

Analytic grade chemicals were used in this study. Thirty percent (30% v/v) ethanol formulated from absolute ethanol (99.7% pure), having Lot # SZBB2160V, was consumed at a concentration of 2 g/kg body weight, Vitamin E 105** **IU/day (DL-α-tocopherol acetate), Ketamine/Xylazine were procured from Sigma-Aldrich Co., USA. Estradiol valerate (Ovlogyn 5 mg/mL ampule obtained from Zafa Pharmaceuticals), sesame oil (Marhaba), metformin tablet 250 mg/kg/day from Martin Dow Pharmaceuticals. Petroleum ether, sulfuric acid (concentrated), ferric chloride, formalin, glacial acetic acid, potassium hydroxide, Mayer's and Dragendorff's reagents, sodium hydroxide, HCl 1%, conjugate solutions for estradiol and testosterone, stop solution, cholesterol reagent, tetramethylbenzidine (TMB), Na_2_CO_3_ (30 g/L), and Horseradish peroxidase (HRP) conjugate reagent were acquired from Sigma (St Louis, MO, USA). Folin–Ciocalteu reagent and gallic acid were procured from Merck (Darmstadt, Germany). Wright-Giemsa stain was obtained from China. Olive oil was procured from Saeed Ghani (Pvt) Ltd., Pakistan. *P. amygdalas* and *J. regia* oil Mixture 4 mL/kg/day was used.

### 2.3. Preliminary Phytochemical Screening

Both oils and their mixture were subjected to preliminary phytochemical screening using standard procedures employed with slight modifications [41–43].

### 2.4. Total Phenolic Content (TPC) Estimation

The TPC were determined using Folin–Ciocalteu reagent as described by Lee et al. [44], and the results (*n* = 3) were expressed as milligram gallic acid equivalent per gram extract (mg GAE/g extract).

### 2.5. GCMS Analysis

The GCMS analysis was implemented in accordance with a published method [45]. GCMS-QP2020 NX-system with autosampler (Shimadzu, Japan) was used. 0.3 μL sample solution was introduced to Rtx-5MS column (30 m × 0.25 mm × 0.25 μm). As a carrier gas, helium was used at 1.0 mL/min. The mass spectra were observed from 40 to 1000 m/z and compared with standard available in spectrum libraries.

### 2.6. Acute Oral Toxicity

Acute oral toxicity of *P. amygdalas*, *J. regia*, and oil mixture was conducted in accordance with OECD guidelines [46]. Both genders' rats were used, provided distilled water, and fed *ad libitum*. Fifty rats were randomly divided into 10 groups (*n* = 5). A single dose of each sample orally; *P. amygdalas* oil, *J. regia* oil, and oil mixture (1:1) was given to rats (*n* = 5) at a dose of (100, 1000, and 2000 mg/kg bw) against control. The rats were inspected for 48 h for toxicity signs such as behavioral changes and mortality [47, 48].

### 2.7. Generational Fertility Study

#### 2.7.1. Animals

Both sexes mature and healthy Wistar rats were used and were procured from animal house, International Center for Chemical and Biological Sciences (ICCBS), and University of Karachi (UoK). They were kept in ventilated cages in standard temperature settings (25–30°C) with a 12-h light and dark cycle. Pelleted rodent food was given to animals. The animals were kept in animal house, Department of Pharmacology, UoK. Twelve-week-old male rats (140–160** **g) and 14-week-old female rats (160–180 g) were given a week of acclimatization period. The method fulfills the guidelines on the proper use and care of laboratory animals [49]. The design and experimental protocol were validated by the BASR (Board of Advanced Studies and Research), ASRB/No./05071/Pharm {Resolution No. 10(P)07}, University of Karachi.

#### 2.7.2. Experimental Protocol for Generation Study

The study was employed over a 7-month duration. A revised method of Vohra and Khera [50] was used. The oil mixture consists of *P. amygdalas* oil and *J. regia* oil in equal parts (1:1). Eighteen rat pairs (*n* = 36, *F*_0_ generation) were placed in cages separately.

For the fertility study, sexually matured 12-week-old male rats (155.42 ± 1.89 g) and 14-week-old female rats (169.1 ± 2.16 g) were kept isolated and inspected for any injury/disease symptom. After an acclimatization week, experimental groups were designed. Three groups comprised male rats, each having (*n* = 6) six rats, while the other three groups comprised female rats (*n* = 6).

In male groups, Group I (control animals) were untreated and given distilled water daily; animals in Group II were given a low dose of oil mixture; 2 mL/kg/day; while Group III animals were given oil mixture at a high dose of 4 mL/kg/day [51]. For the female group, a similar protocol was followed. All rats were treated before mating (Pre-Cohabitation) by gastric gavage for 30 days between 14:00 and 16:00 h. The female rats were exposed to the bedding of male rat to adjust their estrus cycle [52]. Each male rat during mating was individually paired with female rat from each group separately (1:1) for 7 days. Inseminated females were parted from males and placed in cages. The doses of oil mixture were continuously given to female rats throughout the cohabitation phase (21 days), gestation (21 days), delivery, and lactation till day 22 of postnatal period. Gestated female rats were allowed to deliver pups (*F*_1_ generation) naturally.

Afterward, female fertility indices were estimated. The offsprings were observed for abnormalities on postnatal days 0, 4, 7, 14, and 21. All *F*_0_ male/female parental animals were assessed for toxicity signs twice daily.

For the *F*_1_-generation, pups were raised up to 4 weeks, and the same doses of oil mixture were administered according to their body weight, throughout their growth and up to breeding. Following another 4 weeks, rats were permitted mating by forming pairs on a 1:1 basis, in the same treatment group, for seven consecutive days, avoiding sibling mating. The presence of vaginal plug confirmed the gestation of each female rat. Gestated female rats of *F*_1_ generation were parted from male rats and individually placed. Oil mixture was given continuously during all phases as mentioned above, until the weaning of *F*_2_ generation pups. Same protocol of the *F*_0_ generation was implemented for both generations (*F*_1_ and *F*_2_). Gestated rats were allowed to deliver pups naturally (*F*_2_ generation). For *F*_1_ generation female rats' fertility assessment, the stated procedure for assessing fertility indices was used as described by Vohra and Khera [50].

Afterwards, all animals (male and female) were anesthetized with Ketamine (10 mg/kg) and Xylazine (5 mg/kg) intraperitoneally [53], to lessen the suffering of rats. The rats were euthanized by cervical dislocation and blood samples were reserved from the aorta for assessment.

#### 2.7.3. Reproductive Performance of Female Rats

The reproductive performance parameters were mentioned in the form of weights, indices, and ratios that assessed complete stages from conception till weaning of pups [50, 54]. These parameters were calculated as follows:

(1) $$\begin{array}{c} \text{Fertility index}=\left(\frac{\text{Females giving birth}}{\text{females lived together}}\right)\times 100,\\ \text{Litter size}=\frac{\text{Number of pups}}{\text{Number of gestated females}},\\ \text{Live birth index}=\left(\frac{\text{Day }0\text{ alive pups}}{\text{Pups born}}\right)\times 100,\\ \text{Survival index}-4\text{ days}=\left(\frac{\text{Pups alive on day }4}{\text{day }0\text{ alive pups}}\right)\times 100,\\ \text{Survival index}-21\text{ days}=\left(\frac{\text{Alive pups on day }21}{\text{day }4\text{ alive pups}}\right)\times 100. \end{array}$$

#### 2.7.4. Assessment of Blood Parameters

After delivering pups, all animals were anesthetized with Xylazine and Ketamine. Blood samples were withdrawn for biochemical assessment and reserved under aseptic conditions in B-Ject gel clot activator vacuum tubes for hormonal estimation (FSH, LH, testosterone, and estradiol [55, 56] were analyzed via ELISA Roche Diagnostics, Basil). Similarly, GPx and SOD levels were measured by the kit attained from Glory Science Co., Ltd. (Glory Science Co.2022#45).

#### 2.7.5. Histopathological Examination

Ovaries from *F*_1_ generation animals were collected followed by fixing for 48 h in 10% neutral-buffered formalin, embedded in paraffin, partitioned, and stained with hematoxylin and Eosin (H&E) then visualized and compared in groups under a microscope (Bio Base XS-208, China) [57].

### 2.8. Infertility Models

#### 2.8.1. Ethanol-Induced Male Infertility Model

In the fertility study, healthy Wistar male rats weighing 160–200 g were used. The animals were kept in ventilated cages under standard temperature settings (25–30°C) with a 12-h light and dark cycle and were placed in an animal house, Department of Pharmacology, University of Karachi. A modified method by Oremosu et al. was employed to induce infertility in male rats by ethanol [58]. Animals were separated randomly into four groups (*n* = 6). Group I control animals were untreated and daily given distilled water; Group II animals were given ethanol 2 g/kg/bodyweight only, which served as negative control; Group III animals were given an oil mixture of 4 mL/kg/day [51], while Group IV animals were given Vitamin E at a dose of 105** **IU/day [59] as this group served as a positive control. The adopted method of dosing for all groups was gastric gavage. All groups were pretreated with 2 g/kg ethanol for up to 8 weeks daily except control.

Afterward, all animals were deeply anesthetized as described previously and blood samples were reserved for biochemical testing [53]. All blood samples were assessed for hematological parameters (Hb, WBCs, Platelets) via Sysmex KX-21 hematological analyzer [60], Total lipid profile (LDL, VLDL, HDL, Triglycerides, Cholesterol), hormonal estimation (FSH, LH, testosterone, estradiol, and prolactin) via ELISA Roche Diagnostics, Basil [61]. GPx and SOD were assessed by kit obtained from Glory Science Co., Ltd. (Glory Science Co., 2022#45).

For assessing male rats' fertility, the process described by Quadri and Yakubu [62] was adopted to prepare semen obtained from epididymis. The semen was drawn (10 μL) from the caudal epididymis by a capillary tube and placed in petri dishes containing 1 mL, 0.1 M phosphate buffer having a pH of 7.4. The sperm cells were allowed to separate in solution for 10 min at 37°C. Later, the semen pH, motility, volume, viability, and count were estimated by using standard methods. For histopathological evaluation, the testes of the selected rats were removed and processed as above [57].

#### 2.8.2. Estradiol-Induced Female Infertility Model

The female rats were checked daily for ovarian cycle throughout the experimental period by vaginal smear cytology in order to evaluate the estrous cycle phase. The estrous cycle in rats lasts around 4 days usually. The lavage fluid was placed on a glass slide, followed by air-drying, then stained by applying the Wright Giemsa stain. The cytology and estrous cycle stage was determined by employing the method as stated by Nagao et al. [63]. Only those rats having 4–5 days of regular estrous cycles were used in the study. Persistent vaginal cornification and abnormal estrous cycles were considered early signs of ovarian cysts and PCOS induction [64].

To induce infertility in female rats by PCOS, a modified method by Venegas et al. was employed [65]. Animals (*n* = 6) were randomly divided into four experimental groups. All groups were dosed intramuscularly with 1 mg/kg/day (0.1 mL/day) estradiol valerate, dissolved in 0.05 mL sesame oil, except control for 60 days.

Following PCOS induction, the protocol of dosing for 21 days was followed as; Group I control animals were untreated and daily given distilled water; Group II animals were given 0.1 mL estradiol valerate only, that served as negative control; Group III animals were given oil mixture (4 mL/kg/day) [51]; Group IV animals served as positive control and were given metformin at a dose of 250 mg/kg bw per oral daily [66]. The animals were dosed for up to 60 days.

Afterward, all animals were anesthetized as described before [53] and blood samples were withdrawn under aseptic conditions for hematological parameters (Hb, WBCs, platelets) via Sysmex KX-21 hematological analyzer [60], Glucose and HbA1c level, total lipid profile (VLDL, HDL, triglycerides, LDL, cholesterol), hormonal estimation (FSH, testosterone, progesterone, LH) via ELISA Roche Diagnostics, Basil [61]. GPx and SOD were analyzed by the kit obtained from Glory Science Co., Ltd. (Glory Science Co., 2022#45). For histopathological examination, the ovaries of the selected rats of *F*_1_ generation were removed, cleaned, and processed as above [57].

### 2.9. Statistical Methods/Analysis

Data were expressed as mean ± SD (standard deviation). Data for multiple comparisons were done by one-way analysis of variance (ANOVA) with Bonferroni post hoc test using SPSS software statistical program (SPSS for Windows version 20, USA). All results were assumed to be statistically significant if *p* < 0.05, and highly significant if *p* < 0.01. Graph Pad Prism 7 (San Diego, CA) was used to create all graphs.

## 3. Results

### 3.1. Preliminary Phytochemical Screening

The qualitative phytochemical analysis (Table 1) reveals the presence of phenols and flavonoids in all three samples, while glycosides, phlobatannins and terpenoids were present in *P. amygdalas* oil and in oil mixture. Alkaloids and Tannins were absent in all samples.

### 3.2. TPC Analysis

TPC was evaluated by the Folin–Ciocalteu (F–C) method employing standard gallic acid. Absorbance values were assessed to construct the calibration curve at various concentrations of gallic acid. TPC was designated as gallic acid equivalents [67] in dry weight per gram of sample (mg/g) (Table 2).

### 3.3. GCMS Analysis

**Figure 1:**
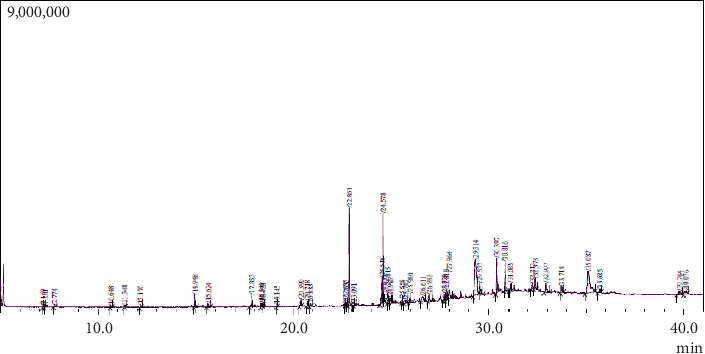
GC-MS chromatogram of oil mixture.

The chemical composition of *P. amygdalas* and *J. regia* oil mixture was obtained by GCMS (Figure 1) and 42 compounds from various classes were obtained as revealed in Table 3. The most concentrated compounds are listed as sterols and fatty acid derivatives. These include lathosterol (15.30%), campesterol (12.85), palmitic acid (10.25%), elaidic acid (9.06%), stigmastanol (5.29%), and trans-squalene (4.84%). The less concentrated compounds include fatty acid derivatives such as stearic acid (2.66%), oleic acid (0.96%), eicosadienoic acid (3.58%), lauric acid (1.58%), sterols and related compounds such as Cholestane-triol, and antioxidants like Gamma tocopherol (1.07%), vitamin E (2.59%), and Thunbergol (1.59%).

### 3.4. Acute Oral Toxicity

Regarding the safety of *P. amygdalas* and *J. regia* plants, acute toxicity results showed that they are harmless at low concentrations but lethal at higher doses (2000 mg/kg each). Acute toxicity study revealed no signs of toxicity along with no mortality within any group of rats. However, some dose-dependent behavioral modifications were observed in the high-dosed groups of *J. regia*. Acute toxicity (LD_50_) results by using Karber Method are stated below (Table 4).

### 3.5. Generational Fertility Study

#### 3.5.1. Breeding Rate

The effect of *P. amygdalas* and *J. regia* oil mixture on the breeding of rats in both *F*_1_ and *F*_2_ generation is summarized in Table 5. In each generation, pups were counted and examined for congenital abnormalities. No abnormality was observed in any group. One-way ANOVA of the oil mixture treatment reveals significant increase in number of pups/litter of rats in treated groups in *F*_2_ generation compared to the control.

Post hoc analysis by the Bonferroni test reveals that statistically significant differences were observed at high-dose administration of oil mixture in rat groups of *F*_2_ generation as compared to the control (df 5.30; F 2.72; *p* < 0.05). Additionally, insignificant differences were found among *F*_1_ and *F*_2_ generations.

#### 3.5.2. Reproductive Performance of Female Rats

The reproductive performance was evaluated by observing numerous parameters that included fertility index, litter size, and live birth index. Insignificant changes were observed in all treated groups indices (*p* > 0.05). All treated groups showed improved parameters, as the control; however, a significant improved effect in litter size was noted in both generations' treated groups of rats (*p* < 0.05) against the control group, as mentioned in Table 6.

#### 3.5.3. Blood Parameter Assessments

##### 3.5.3.1. Hormonal Analysis

**Figure 2:**
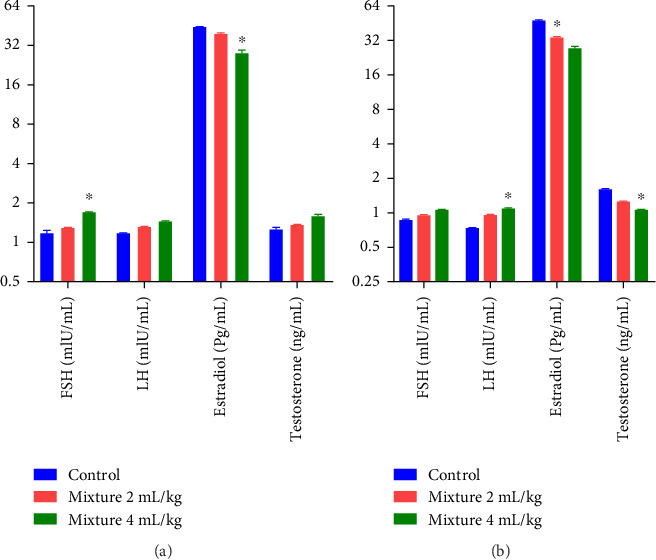
Effect of oil mixture on hormonal parameters as compared to control: male (a); female (b). *n* = 6, Mean ± SEM; ^∗^*p* < 0.05 significant, ^∗∗^*p* < 0.01 highly significant as compared to control.

The effect of *P. amygdalas* and *J. regia* oil mixture on hormonal parameters (FSH, testosterone, LH, and Estradiol) in low and high doses concerning gender is summarized in Figure 2.

One-way ANOVA reveals that significantly increased FSH levels were observed in male rats (*p* < 0.05), while in female rats, significantly increased LH levels, and decreased Testosterone levels were observed (*p* < 0.05) in comparison to the control.

Post hoc analysis by the Bonferroni test reveals that statistically significant differences in FSH (df 5.30; F 44.30; *p* < 0.05) and estradiol (df 5.30; F 48.94; *p* < 0.05) were found in male rats after high-dose oil mixture administration as compared to control. However, female rats exhibited statistically significant differences in LH (df 5.30; F 205.35; *p* < 0.05) and testosterone (df 5.30; F 191.1; *p* < 0.05) as compared to the control. Additionally, a significant effect was found on estradiol at low-dose oil mixture treatment (df 5.30; F 115.2; *p* < 0.05) in female animals as compared to the control.

##### 3.5.3.2. Antioxidant Activity by SOD and GPx Assessment

**Figure 3:**
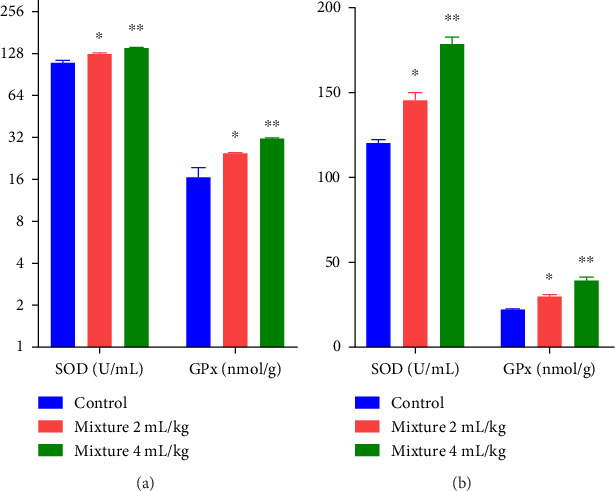
Effect of oil mixture on oxidative parameters as compared to control: male (a); female (b). *n* = 6, Mean ± SEM; ^∗^*p* < 0.05 significant, ^∗∗^*p* < 0.01 highly significant as compared to control.

The effect of *P. amygdalas* and *J. regia* oil Mixture on oxidative parameters in low and high doses is summarized in Figure 3. Parameters like SOD and GPx were assessed in the plasma of control and treated rats of both genders.

One-way ANOVA reveals that in male rats, there was a significant increase in SOD (*p* < 0.01) and GPx (*p* < 0.01) as compared to control group. Similarly, females also exhibited a significant increase in SOD (*p* < 0.01) and GPx (*p* < 0.01) levels as compared to the control group.

Post hoc analysis by the Bonferroni test exposed a statistically highly significant effect of the administration of high-dose oil mixture on SOD in male (df 5.30; F 20.83; *p* < 0.01) and female (df 5.30; F 68.41; *p* < 0.01) animals. Similarly, a highly significant effect was also obtained on GPx levels at high-dose treatment in male (df 5.30; F 18.74; *p* < 0.01) and female rats (df 5.30; F 50.32; *p* < 0.01) in comparison to the control. A statistically significant effect was also observed on SOD and GPx at low-dose treatment. This result suggests a dose-dependent relationship between oil mixture and the oxidative parameters.

##### 3.5.3.3. Histopathological Evaluation

**Figure 4:**
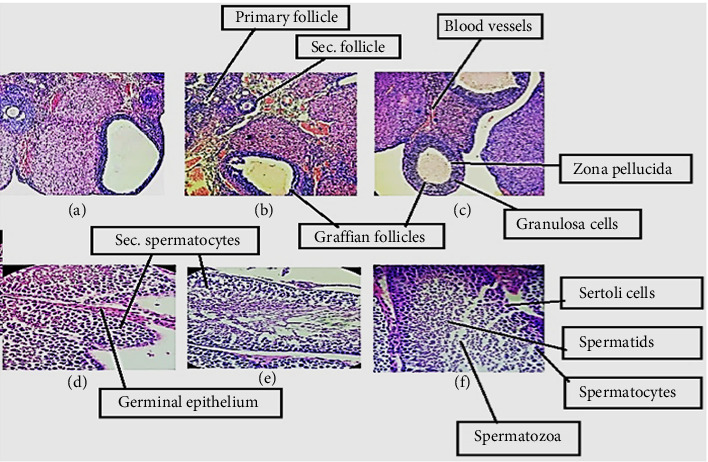
Effect of oil mixture on histopathological parameters. (a) Micrograph rat ovary of control (10x). (b) Micrograph rat ovary low-dose oil mixture–treated (20x). (c) Micrograph rat ovary high-dose oil mixture–treated (20x). (d) Micrograph rat testis of control (20x). (e) Micrograph rat testis low-dose oil mixture–treated (40x). (f) Micrograph rat testis high-dose oil mixture–treated (40x).

Figure 4 displays the histological observation of ovaries of the control animals (a) revealed several healthy follicles at progressive stages of development and fresh corpus luteum. There were also few atretic follicles. Moreover, low-dose-treated animals of oil mixture (b) show healthy primary and secondary follicles observed frequently with mature stages of Graafian follicles. Animals treated with high-dose oil mixture (c) show multiple preantral and Graafian follicles with active or fresh corpus luteum than the control.

In testes (d) sections, we found normal epithelial thickness, spermatogenesis and germ cells in the control section. The spermatogenic cells constitute a thick layer. Low-dose oil mixture–treated testes (e) seemed similar to the control sections; however, high-dose oil mixture–treated (f) testis showed improved testicular structure and spermatogenesis as compared to the control.

### 3.6. Infertility Models

#### 3.6.1. Ethanol-Induced Male Infertility Model

The parameters like sperm motility, count, pH, volume and viability are critical indices of male fertility as they are crucial markers in testicular spermatogenesis. These parameters were improved significantly (*p* < 0.05) in oil mixture–treated group as compared to the control group, as displayed in Table 7.

**Figure 5:**
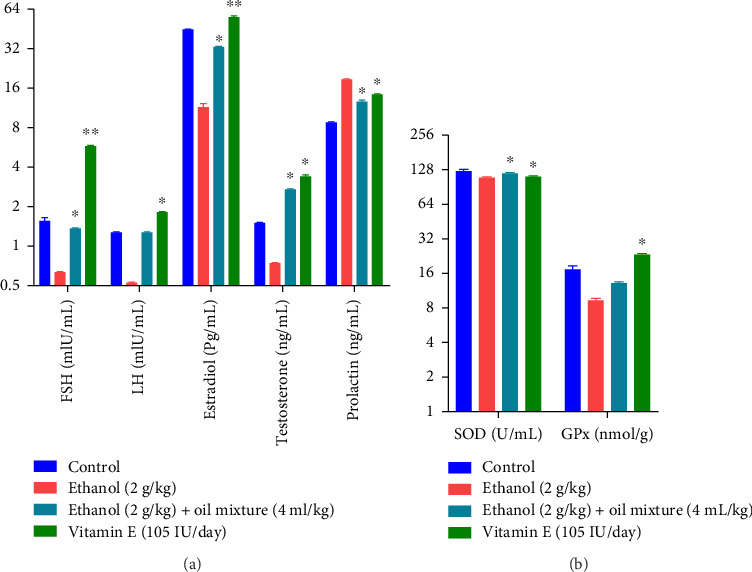
Effect of oil mixture on the hormonal and oxidative parameters as compared to negative control. *n* = 6, Mean ± SEM; ^∗^*p* < 0.05 significant, ^∗∗^*p* < 0.01 highly significant as compared to control.

The effect of oil mixture on hormonal and oxidative markers in male rats for 60-days (8 weeks) dosing is summarized in Figure 5. One-way ANOVA reveals a significant increase in FSH (df 3.20; F 3320; *p* < 0.05), LH (df 3.20; F 419.2; *p* < 0.05), estradiol (df 3.20; F 495; *p* < 0.05), testosterone (df 3.20; F 1379; *p* < 0.05), and a significant decrease in prolactin (df 3.20; F 367.2; *p* < 0.05) as compared to the negative control group.

On Oxidative parameters, one-way ANOVA reveals that the oil mixture produced a significantly improved effect on SOD (df 3.20; F 3.55; *p* < 0.05) in comparison to the negative control group.

**Figure 6:**
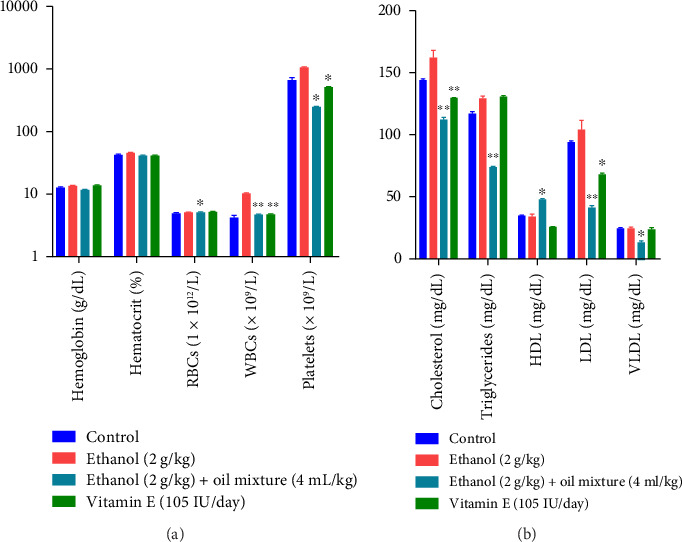
Effect of oil mixture on the hematological and biochemical parameters as compared to negative control. *n* = 6, Mean ± SEM; ^∗^*p* < 0.05 significant, ^∗∗^*p* < 0.01 highly significant as compared to control.

The effect of the oil mixture on hematological and biochemical parameters in rats for 60-day dosing is summarized in Figure 6. One-way ANOVA reveals that the oil Mixture produced a significantly raised effect on RBCs (df 3.20; F 3.52; *p* < 0.05) as compared to the negative control group. Moreover, a highly significant decreased effect on WBCs was observed by oil mixture (df 3.20; F 175.8; *p* < 0.01) that is similar to the standard vitamin E as compared to the negative control group. Additionally, the Oil mixture produced significant decrease in platelets (df 3.20; F 154.6; *p* < 0.05), similar to Standard Vit E as compared to the negative control group.

On Biochemical Parameters, one-way ANOVA reveals that the oil mixture produced a highly significant effect by decreasing Cholesterol, Triglycerides and LDL (df 3.20; F 44.37; 271.7; 52.7; *p* < 0.01), respectively, as compared to the negative control group. Additionally, VLDL was reduced significantly by oil Mixture (df 3.20; F 35.05; *p* < 0.05) in comparison to the negative control group. However, the oil mixture increased HDL (df 3.20; F 52.3; *p* < 0.05) in comparison to the negative control group.

**Figure 7:**
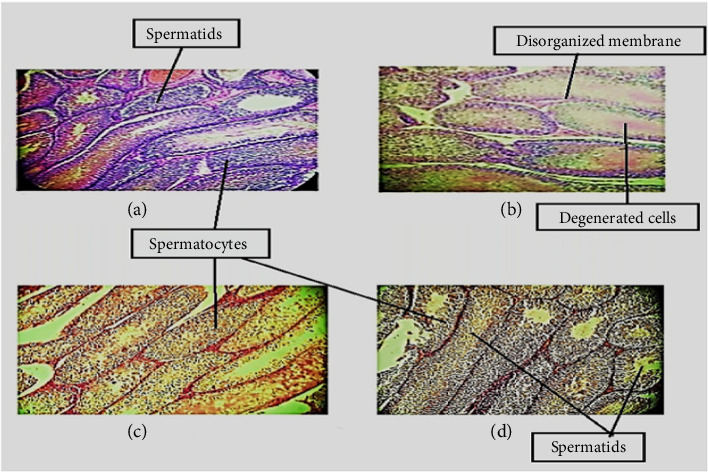
Effect of oil mixture on histopathological parameters. (a) Micrograph rat testis of control (20x). (b) Micrograph, rat testis ethanol-treated (20x). (c) Micrograph, rat testis treated with ethanol and oil mixture (20x). (d) Micrograph, rat testis treated with ethanol and vitamin E (20x).

Figure 7 shows the histology of rat's testis from different groups. Group (a) sections of control rats showed typical architecture of testicular tubules having numerous germinal cells with spermatozoa filled lumen. The Group (b) ethanol-treated rats exhibited loss of germinal cells, detachment and severe degeneration of seminiferous tubules which presents disorganized and irregular membranes. Sections of testes from Group (c) oil mixture–treated rats showed partial loss of germinal cells and normal presence of spermatogenic epithelium, that presents a regular arrangement and fullness of spermatogenic cells in comparison with ethanol-treated rats. Sections of testes from Group (d) animals treated with Vit E showed organized membranes. Primary and secondary spermatids were noticed in the central region of seminiferous tubular lumen. Enhanced production of spermatozoa and spermatids were observed as compared to the negative control.

#### 3.6.2. Estradiol-Induced Female Infertility Model

**Figure 8:**
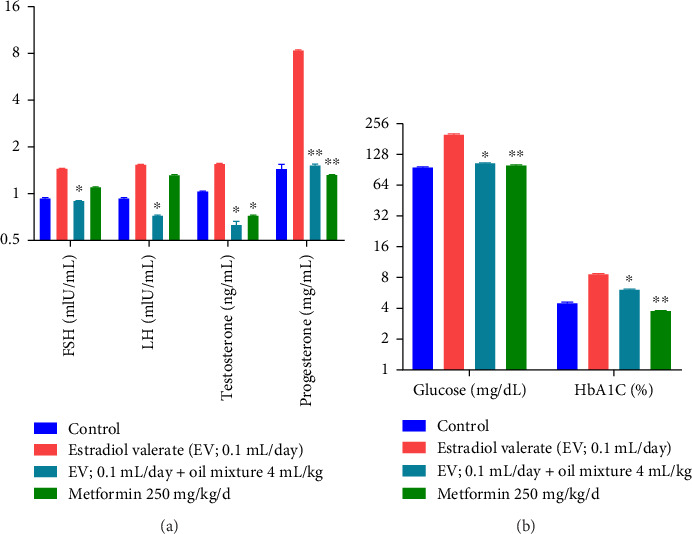
Effect of oil mixture on hormonal and blood glucose parameters as compared to negative control. *n* = 6, Mean ± SEM; ^∗^*p* < 0.05 significant, ^∗∗^*p* < 0.01 highly significant as compared to control.

The oil mixture's effect on rats' hormonal and blood glucose parameters is summarized in Figure 8. One-way ANOVA reveals significant declined levels of LH, FSH, and testosterone (df 3.20; F 370.2; 883.1; 330.5; *p* < 0.05), respectively, as compared to the negative control group. Additionally, it was revealed that Progesterone is highly significantly decreased by oil mixture (df 3.20; F 1973.2; *p* < 0.01), similar to metformin, compared to the negative control group.

On glucose parameters, the oil mixture produced significant effects in comparison to the negative control group. One-way ANOVA reveals a significant reduction in glucose and HbA1c levels (df 3.20; F 1432.4; 520.2; *p* < 0.05), respectively, compared to the negative control group.

**Figure 9:**
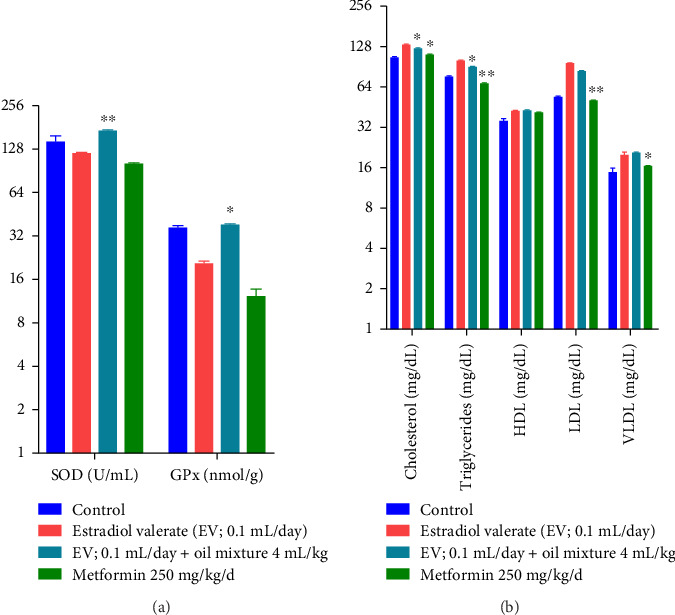
Effect of oil mixture on oxidative and biochemical parameters as compared to negative control. *n* = 6, Mean ± SEM; ^∗^*p* < 0.05 significant, ^∗∗^*p* < 0.01 highly significant as compared to control.

The effect of the oil mixture on the oxidative and biochemical markers in rats is summarized in Figure 9. One-way ANOVA reveals a highly significant SOD level increase by oil mixture (df 3.20; F 19.11; *p* < 0.01) in comparison to the negative control group. Additionally, the level of GPx was significantly increased (df 3.20; F 115.1; *p* < 0.05) as compared to the negative control group.

On biochemical parameters, one-way ANOVA reveals a significant decrease in cholesterol and triglyceride levels (df 3.20; F 114; 196.1; *p* < 0.05), respectively, compared to the negative control group. Insignificant results on HDL, LDL, and VLDL (df 3.20; F 12.23; 1492.4; 11.9; *p* > 0.05), respectively, were obtained from the oil mixture group as compared to the negative control group.

**Figure 10:**
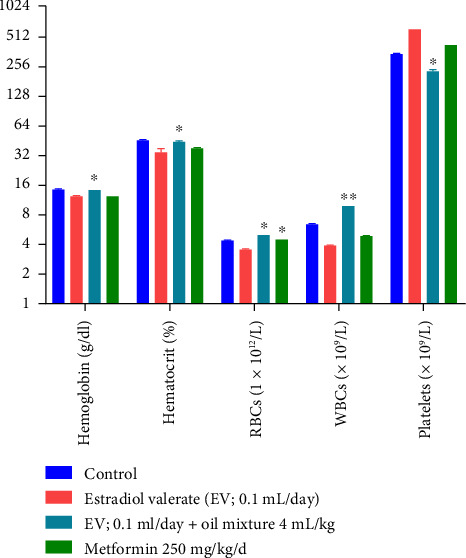
Effect of oil mixture on hematological parameters as compared to negative control. *n* = 6, Mean ± SEM; ^∗^*p* < 0.05 significant, ^∗∗^*p* < 0.01 highly significant as compared to control.

The effect of oil mixture on hematological parameters in rats is summarized in Figure 10. One-way ANOVA reveals a significant increase in hemoglobin and hematocrit levels by oil mixture (df 3.20; F 167.1; 10.43; *p* < 0.05) in comparison to the negative control group. However, a significant decrease was also found in the platelets level (df 3.20; F 15,820; *p* < 0.05) as compared to the negative control. Moreover, the levels of WBCs were highly significantly increased (df 3.20; F 1408; *p* < 0.01) as compared to negative control animals.

**Figure 11:**
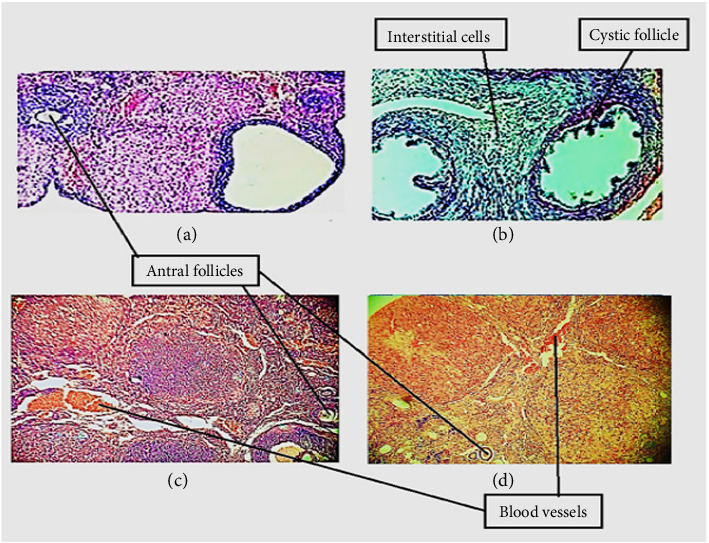
Effect of oil mixture on histopathological parameters. (a) Micrograph rat ovary of control (20x). (b) Micrograph rat ovary treated with estradiol (40x). (c) Micrograph rat ovary treated with estradiol and oil mixture (20x). (d) Micrograph rat ovary treated with estradiol and metformin (20x).

Figure 11 shows the histology of ovaries from different groups of rats. The control group sections (a) showed corpus luteal cells, mature follicles (Graafian follicles) along with granulosa cells, and various primary and secondary follicles. The cell structures were typical. Group (b); Estradiol-treated rat ovaries showed an increase in cystic follicles, absence of corpus luteum, thin granular and thick theca layer with increased interstitial cells. Group (c) oil mixture–treated group showed normal architecture with regenerated corpus luteum along with augmented preantral and Graafian follicles. Group (d) metformin-treated group showed normal architecture with regenerated corpus luteum in various phases. The follicle counts were significantly elevated in metformin-treated animals.

## 4. Discussion

The study elaborates *J. regia* and *P. amygdalas* mixture's role as fertility-enhancing agents. In this regard, the current study represented the presence of flavonoids, glycosides, phenols, saponins, phlobatannins, and terpenoids. Additionally, the quantitative data represents the presence of 0.02 mg/g GAE/TPC. Phenolic compounds are commonly associated with anti-inflammatory, antioxidant, antiatherogenic, and have anticancer properties. It was reported that high phenolic contents are present in the nut and skin of *P. amygdalas* and *J. regia* [68, 69]. The presence of tocopherols, squalene, and polyphenols strongly correlated to the *J. regia* oil antioxidant capacity [70, 71]. Further, GCMS analysis of the oil mixture depicted the presence of sterols and PUFAs in higher concentrations especially lathosterol, campesterol, palmitic acid, elaidic acid, stigmastanol, gamma-tocopherol, etc. (Table 3, Figure 1). It was previously reported that diets rich in antioxidants and PUFAs can help counteract oxidative stress and lipid peroxidative damage [72]. Oil mixture was evaluated for acute toxicity in rats that revealed only one animal was dead with 6.66% mortality up to the dose of 2000 mg/kg/BW. According to Kulkarni et al., *P. amygdalas* oil was nontoxic in acute oral toxicity and was found to be safe up to a dose of 2000 mg/kg in rats [73].

Further fertility-enhancing effects were observed in a bi-generational study. We observed increased pups' production in both *F*_1_ and *F*_2_ generations with both doses of oil mixture, as shown in Table 5. This showed that oil presented its beneficial effects by augmenting the procreativity of the male and female rats. High levels of endogenous hormones could be the reason for infertility in women. Almond oil balance hormonal production and maintain the menstrual cycle and fertility performance in women [74]. Similarly, a study indicated that daily consumption of walnuts improved sperm motility, morphology, and vitality in a group of healthy men and considered it as sperm motility enhancer associated with increased omega-6 and omega-3 PUFAs [75]. *F*_2_ generation pups represented fertility-enhancing effect by increasing litter size and live birth index when compared to control. However, no effects were noticed on viability, mating, and fertility indices on 4 and 21 days. These results are in accordance with Ayokanmi et al., who reported that the fertility-enhancing effect of almond oil is associated with the presence of vitamin E (tocopherol) [76]. Almonds also contain a significant amount of PUFAs as well as zinc, magnesium, vitamin B_6_, and folate that stimulate the hypothalamus to increase testosterone production, which increases sperm production and improves sperm quality. Similarly, *J. regia* contains many PUFAs such as linoleic acid, linolenic acid, and oleic acid [77]. They prevent the formation of dihydrotestosterone by inhibiting 5-α reductase, restoring the estrogen level in postmenopausal women and testosterone level in males [78, 79].

Reproductive hormones are crucial in the growth and regular functioning of the reproductive system. Testosterone and FSH are necessary for augmenting reproductive capabilities in males [80]. Researches displayed that sex hormones are related with spermatogenesis [81]. FSH, LH, testosterone, and estradiol levels were assessed in both gender animals. The findings revealed significantly raised FSH levels in male rats and LH in female rats (Figure 2) by high-dose oil mixture treatment. Testosterone levels were significantly reduced in female rats; it depicts its potential as a fertility-augmenting agent. Additionally, estradiol levels were declined significantly in both sexes of animals. Since FSH/LH elevation is essential for ovulation instigated by LH-releasing hormone, depends on estradiol levels [82]. Our results suggest that oil mixture significantly improved FSH levels by stabilizing estradiol and enhancing fertility in higher doses, possibly owing to the improved pituitary sensitivity to gonadotropin-releasing hormone (GnRH) [83]. Our findings are in agreement with Bostani et al., who depicted that *J. regia* raised sex hormones effectively in rats [78]. *J. regia* possess α-linolenic acid that forms arachidonic acid, which serves as a precursor for prostaglandins. Prostaglandins are essential in ovulation and testicular steroidogenesis [38]. Similarly, *P. amygdalas* oil improved FSH levels in higher doses by stabilizing the estradiol levels and augmenting fertility [84].

In the pathogenesis of reproductive performances and several inflammatory diseases, oxidative stress is critically important [85]. Reactive oxygen species can serve as significant signaling molecules in physiological processes, but its uncontrolled excess levels can also facilitate pathological processes such as infertility. SOD and glutathione functions as physiological antioxidants, maestro of immune system and the principal detoxifiers [86]; they shield eggs during folliculogenesis from damage, triggered by oxidative stress in females and improve sperm maturation, motility, and count in males [7]. The present study displayed augmented glutathione and SOD levels by oil mixture consumption in both genders of rats that affirmed the antioxidant potential of the oil mixture (Figure 3). Current glutathione results depicted the maximum correlation among antioxidant potential and PUFAs content with polyphenol's presence in an oil mixture. These results are in-agreement with Truong et al., who investigated the fertility role of procyanidins in the skin of *Prunus amygdalas* as an antioxidant compound [87]. Similarly, Miao et al. exhibited that *J. regia* oil significantly enhanced the antioxidant potential by restoring SOD and glutathione levels while reducing the inflammatory factors [88]. The above findings demonstrated that combined treatment with *P. amygdalas* and *J. regia* oil mixture improves fertility in both genders of rats.

To determine the further protective role of the oil mixture in male/female fertility, we designed an ethanol-induced male infertility model and estradiol-induced female infertility model. Alcohol has deleterious effects on reproductive system [89]. Several preclinical studies have been used to establish male infertility models to explore the therapeutic potential of different therapeutic medicines [90, 91]. Some studies highlighted the deleterious effects of alcohol on male sexual functions [92, 93].

The current study explored the beneficial effect of an oil mixture against ethanol-induced male infertility model that revealed ethanol successfully induced infertility in male rats as ethanol-induced negative control groups showed altered sex hormone levels. Results of LH, FSH, and testosterone showed that treatment with ethanol suppressed sex hormones production in experimental groups in comparison to control and oil-treated groups (Figure 5). Various researchers have investigated the efficacy of their treatments against ethanol and enlightened ethanol-induced toxic effects on reproductive organs and sperm profile, leading to azoospermia [94–97]. The current study showed improved LH, FSH, and testosterone levels as the oil mixture is rich in omega 3 and 6 fatty acids. A previous randomized controlled trial reported that supplementing a Western-style diet with hazelnuts, almonds, and walnuts recovers sperm quality parameters in healthy aged men [98]. Estradiol levels were significantly decreased, while prolactin levels were enhanced in comparison to control group. It was earlier reported that ethanol consumption disturbs the male hormonal balance and alters prolactin and estradiol levels [99]. However, the administration of the oil mixture successfully restored these hormonal alterations and improved testicular activity. This potential of the *P. amygdalas* and *J. regia* oil mixture could be due to its antioxidant properties and presence of PUFAs, fiber, riboflavin, niacin, β-sitosterol, and stigma sterol; alpha-tocopherol and polyphenols as revealed by the study of King et al. [100]. High levels of these PUFAs and polyphenols are more desirable because of their health benefits in metabolism, brain plasticity, and reproduction [101, 102]. Rehman et al. reported that the *P. amygdalas* oil benefits sperm morphology and improves sex hormone disturbances [103]. Similarly, Anwar et al. reported that this potential of *J. regia* oil could be due to its antioxidant properties and the presence of PUFAs and polyphenols [104].

SOD and glutathione levels were found to be elevated by oil mixture consumption (Figure 5). The oil mixture contains *P. amygdalas* oil and *J. regia* oil, which are rich in PUFAs and known to have anti-inflammatory effects by decreasing cytokines and free radical formation [105]. PUFAs within cellular membranes are essential to maintain the characteristics of the lipid bilayer. Spermatozoa membrane lipids are essential for the flexibility and fluidity of spermatozoa as well as for successful fertilization [106]. Liu et al. reported that *J. regia* oil consumption significantly reduces ethanol-induced liver damage and increases the activity of antioxidant enzymes [107, 108].

Blood indices (RBCs, MCV, MCH, and MCHC) are particularly important for diagnosing anemia in most animals. It was demonstrated in the present study that ethanol affects hematological parameters deleteriously. This finding revealed that ethanol administration to rats raised total WBCs and platelet count significantly (Figure 6). Results are in line with Osonuga et al. who reported ethanol altered hematological data and significantly raised WBC count [109]. Current results showed that the oil mixture successfully restored RBCs, WBCs, and platelet counts. The study discovered that oil mixture stimulates the hemopoietic system and restores WBCs activity by modifying their count toward normal. As a result, it may be advised for those who are immunocompromised, have bone marrow loss, or have anemia. In addition, our results are in agreement with Koriem and El-Attar that discovered *P. amygdalas* supplementation has successfully restored blood parameters, especially leukocytes and red blood cells [110]. Moreover, our results are in line with Alexander et al. who discovered that Nigerian walnut has a positive effect on the hematopoietic activity of Swiss Wistar rats [111].

The results from ethanol-treated animals displayed increased TC, LDL, VLDL, and TG levels. Moreover, a decrease in HDL levels was also found in ethanol-induced rats. This may indicate an alteration in the permeability of hepatic cells [112]. The increase in total cholesterol level may be attributed to the liver bile ducts blockage, causing a reduction of cholesterol secretion in the duodenum [113]. Exposure to ethanol shows a marked perturbation in the metabolism of lipids leading to reduced semen quality, count, and motility [114]. Oil mixture administration significantly restored the levels of HDL and reduced cholesterol, triglyceride, and LDL levels (Figure 6). Probably, this effect is due to the presence of PUFAs and polyphenol contents, as Yanai et al. reported that Omega-3 PUFAs increase HDL [67]. The HDL formation is linked to the catabolism of triglyceride-rich lipoproteins such as VLDL or intermediate-density lipoprotein (IDL). So, increased PUFAs consumption reduces LDL and VLDL and raises HDL. Our results are in accordance with Al-Attar who reported that almond oil has antioxidants that by inhibiting free radicals formation, may delay autoxidation [115].

Histopathological findings revealed that ethanol-treated rats presented degeneration of seminiferous tubules, loss of germinal cells, disorganized and irregular testicular membrane. Sections of testes treated with an oil mixture showed recovery of the germinal cell, seminiferous tubules, and luminal spermatozoa. Akomolafe et al. reported histological irregularities in the testicular tissue and seminiferous tubules of alcohol-treated animals. In addition, they disclosed that the sperm motility and count were reduced in ethanol-treated rats [95]. Result showed oil mixture–treated animals significantly ameliorated all degenerative ethanol changes (Figure 7). These findings are in accordance with Al-Attar et al. who stated that *P. amygdalas* and/or *J. regia* oil, by preventing the lead-induced oxidative stress, contributed to the sustainment of pro/antioxidant balance in the epididymal and testicular environment [115, 116].

To determine the further protective role of the oil mixture in female fertility, we designed an estradiol-induced female infertility model. The typical cause of female infertility is PCOS. PCOS is listed as an endocrine disorder worldwide, affecting 4%–18% of reproductive-age women. Unfortunately, the percentage of women affected by PCOS is much higher in Pakistan, about 52% [117]. In PCOS, it has been studied that there is a disruption in the neuroendocrine system which in turn causes the imbalance of the hypothalamic-pituitary-ovarian axis, leading to gonadotrophins overproduction, that is, LH and FSH. Increased hypothalamic GnRH production favors LH over FSH; therefore, in PCOS, elevated LH/FSH ratio levels were observed [118]. Hyperandrogenism is also one of the critical features of PCO; it is reported to cause excess insulin release by the pancreatic β-cells, decreasing insulin-like growth factors production in the liver [119]. This growth factor produces maturation failure and differentiation of granulosa cells, which induces preantral follicular growth arrest, anovulation and formation of cyst [120]. Oral contraceptives and metformin are also indicated in PCOS management. However, several adverse effects are associated including acne, irregular menstrual periods, breast tenderness, hirsutism, increased body weight, bloating, nausea, and infertility [121, 122]. Since ancient times, most people have used complementary medicines in Pakistan, including natural and herbal ones, to prevent and treat infertility-related issues. *P. amygdalas*, *J. regia* and other nuts have been used in folklore medicine to boost fertility and reproductive parameters. We used estradiol valerate to induce the PCOS model.

Estradiol valerate is a prolong-acting estrogen that induces hypothalamic-pituitary disturbance of gonadotropin-releasing hormone resulting in inappropriate LH release and storage. It favors rapid disturbance in metabolic and hormonal processes resulting in PCOS [123]. Estradiol valerate induces polycystic models in animals through the production of oxidative stress along with activation of the parasympathetic nervous system, associated with morphological, endocrinological, and metabolic changes [124]. The current study reveals that estradiol valerate markedly increases levels of FSH, LH, estradiol, and progesterone. The oil mixture significantly downregulated FSH and LH levels as compared to estradiol valerate–treated PCOS-induced rats (Figure 8). Additionally, the plasma testosterone and progesterone levels were significantly reduced by oil mixture treatment. Previous research explained that excessive testosterone and progesterone contributed to the pathogenesis of PCOS, and downregulation of these hormones may have useful effects in PCOS [125, 126]. These results suggest that oil mixture may serve as a functional food for women for treating hormonal disturbances associated with PCOS due to the presence of PUFAs and phytoestrogens as endorsed by GCMS. Women with PCOS have low estrogen levels, and the presence of phyto-estrogenic compounds in oil is hypothesized to effectively lower androgen levels and enhance female hormonal levels [52].

Hyperinsulinemia and raised glucose levels are typical features of PCOs. Dunaif et al. found that obese PCOs women had significantly enhanced glucose levels during oral glucose tolerance test as compared to ovulatory hyperandrogenic women [127]. Estradiol valerate significantly raised the glucose levels and HbA1c in rats compared to the control (Figure 8). Insulin resistance is considered as the primary reason of glucose metabolic disease, which is considered to be a critical factor in developing PCOs and strongly correlated to concurrent metabolic difficulties [128]. Increased levels of LH and testosterone are released due to hyperinsulinemia that disturbs the uptake and consumption of glucose, resulting in prolonged anovulation [129, 130]. Estradiol valerate is responsible for increasing serum glucose levels [128]. Treatment with oil mixture ameliorated raised amount of glucose levels and HbA1c as compared to the negative control group. Results are in line with Nasiry et al. who reported that the administration of *J. regia* leaf extract has significantly reduced fasting blood sugar (FBS) and HbA1_c_ as compared to control groups [131]. Similarly, Gulati et al. reported that *P. amygdalas* oil administration significantly reduced HbA1c and glucose levels in Asian Indian patients with type II diabetes [132].

The estradiol valerate–treated rats showed decreased SOD and GPx levels, representing oxidative stress. Ghafurniyan et al. reported that estradiol valerate significantly produced the PCOS model by creating free radial formation and decreasing glutathione levels. Oil mixture-treated rats significantly raised the SOD and GPx levels (Figure 9) owing to their immune-boosting antioxidant effect. Our results are in line with Al-Attar, who reported that the *P. amygdalas* oil has an intense potential to prevent oxidative stress by disrupting the free radical-mediated chain reactions, regulating the production of free radicals, as well as preventing the progression of lipid peroxidation [115]. Thus, oil mixture can potentially balance oxidative stress while restoring cellular homeostasis.

In PCOS conditions, lipid peroxidation and reduction in antioxidant enzyme activity lead to an altered total lipid profile [133]. Patients may experience high LDL cholesterol, triglycerides, total cholesterol levels, and low HDL levels [134, 135]. Experimental rats of the current study treated with estradiol valerate exhibited raised amounts of cholesterol, triglycerides, LDL, and VLDL as compared to the control group (Figure 9). Values are in accordance with the previous studies in nonathletic female population that showed raised cholesterol and LDL levels due to the estrogenic component [136]. Similarly, estradiol valerate alter lipid metabolism by affecting the metabolism of lipoproteins in different ways [137]. Kokabiyan et al. also showed increased TG, TC, and LDL in PCOS rats treated with estradiol valerate [138]. Oil mixture administration significantly decreases cholesterol and triglyceride levels. This effect is probably due to PUFAs and polyphenol contents [67]. As it was reported previously that PUFA decrease triglycerides by enhancing β-oxidation, decreasing hepatic lipogenesis as well as hepatic output that may lead to reduction in triglycerides level [139].

We observed significantly decreased effects of estradiol valerate on hematological parameters (Figure 10). Results are in accordance with Barath et al. who reported that estradiol valerate produces a significant decrease in RBC count, hemoglobin, and hematocrit [140]. Supplementation with an oil mixture restored hematological parameters. Our results are in line with Alexander et al. study that revealed Nigerian walnut has an encouraging effect on hematopoietic activities of Wistar rats [111]. Similarly, Koriem and El-Attar reported that *P. amygdalas* supplementation has successfully restored blood parameters, especially leukocytes and red blood cells [110].

The ovarian histopathological evaluation demonstrated significant changes in the negative control group, such as increased follicular wall thickness, distortion, and a raised number of cystic follicles without corpus luteum. Treatment with an oil mixture showed raised counts of primordial follicles, Graafian follicles and corpus luteum (Figure 11). The result showed the preventive effect of the oil mixture by reducing oxidative stress as it is enriched in polyphenols and the presence of PUFAs, which are proven antioxidants [141]. Our results are in accordance with previous research that explored *J. regia* supplementation exhibited a protective antioxidant effect against lead-induced oxidative damage [141, 142].

## 5. Conclusion and Future Prospects

In conclusion, our results demonstrated that administration of *P. amygdalas* and *J. regia* oil mixture (1:1) had valuable effects on fertility processes in both male and female sexes, by augmenting spermatogenesis and ovulation. This could be associated with reduced glucose level in blood, lipid profile, and oxidative stress while restoring sex hormones. Therefore, it is advised that consumption of *P. amygdalas* and *J. regia*, daily, may provide an effective strategy to improve fecundity. Therefore, *P. amygdalas* and *J. regia* oil mixture (1:1) may be a novel candidate to treat metabolic and reproductive disorders in both male and female infertile patients. Further studies on clinical effects and dose-response relationship are needed.

## 6. Strengths and Limitations of Study

Several studies have explored the effects of nuts especially *P. amygdalas* on male reproduction [143, 144]. However, there has been limited research on its combination with *J. regia* oil and its impact on female/male reproductive health. This study represents the first to focus on the antioxidant potential as well as hormone-boosting effects of combination oil, as previous studies primarily focused on estrogenic properties of the individual oils. These findings provide valuable insights into the potential use of almond and walnut oil in female/male reproductive health. The current study further supports the fertility-enhancing effects of the combination oil, demonstrating improved outcomes likely due to its richness in omega-3 and omega-6 fatty acids.

The major limitations in the present study could be addressed in future. First, it focused primarily on the bi-generational study owing to a reduced budget, we restricted up to *F*_2_ generation; in case of more financial resources and facilities, we must go for *F*_3_ and *F*_4_ generations further, to scrutinize mirror images of study outcomes. Secondly, genomic markers will be assessed for exact molecular mechanisms. The study did not address all factors affecting fertility, like programmed cell death or apoptosis, which may affect gametogenesis including spermatogenesis [145].

## Figures and Tables

**Table 1:** Phytochemical analysis of *P. amygdalas*, *J. regia*, and oil mixture.

Phytochemicals	*P. amygdalas*	*J. regia*	Oil mixture
Phlobatannins	+	−	+
Flavonoids	+	+	+
Glycosides	+	−	+
Alkaloids	−	−	−
Saponin	−	+	+
Phenols	+	+	+
Terpenoid	+	−	+
Tannin	−	−	−

*Note:* The presences of phytochemicals are represented as +, while absence denoted as −.

**Table 2:** Total phenolic content in *P. amygdalas*, *J. regia*, and oil mixture.

Sample	Total phenolic content (mg/g GAE)
*P. amygdalas*	0.03
*J. regia*	0.01
Oil mixture	0.02

**Table 3:** GCMS analysis of oil mixture.

S No.	Name of compound	Retention time (min)	Peak area (%)	Molecular weight	Formula
1	Dimethyl benzene	7.169	0.09	106	C_8_H_10_
2	Carbonochloridic acid	7.281	0.15	164	C_7_H_13_ClO_2_
3	m-Xylene (dimethyl benzene)	7.774	0.21	106	C_8_H_10_
4	1,3-Dichlorobenzene	10.64	0.12	146	C_6_H_4_Cl_2_
5	3,7-Dimethylundecane	11.34	0.22	184	C_13_H_28_
6	3,7-Dimethyldecane	12.17	0.19	170	C_12_H_26_
7	Octadecane	14.94	1.29	254	C_18_H_38_
8	4,6-Dimethyldodecane	15.62	0.33	198	C_14_H_3_O
9	Heptadecane	17.85	1.55	240	C_17_H_36_
10	Lauric acid	18.34	0.37	214	C_13_H_26_O_2_
11	Cetane/Hexadecane	18.42	0.18	226	C_16_H_34_
12	Butanoic acid	19.14	0.15	158	C_8_H_14_O_3_
13	Heneicosane	20.39	0.64	296	C_21_H_44_
14	Myristic acid	20.71	0.64	242	C_15_H_30_O_2_
15	Ethanone	22.71	0.3	220	C_14_H_20_O_2_
16	Palmitic acid	22.86	10.25	270	C_17_H_34_O_2_
17	Diethyl heptadecane	23.09	0.15	296	C_21_H_44_
18	Eicosadienoic acid	24.51	3.58	322	C_21_H_38_O_2_
19	Elaidic acid	24.57	9.06	296	C_19_H_36_O_2_
20	Stearic acid	24.81	1.60	298	C_19_H_38_O_2_
21	Eicosenoic acid	24.92	1.42	310	C_20_H_38_O_2_
22	Eicosyl acetate	25.56	0.32	340	C_22_H_44_O_2_
23	1-(1-Heptadecynyl) cyclopentanol	25.73	0.49	320	C_22_H_40_O
24	N,N-Dimethyldecanamide	25.98	1.29	199	C_12_H_25_NO
25	Methyl henicosanoate	26.61	1.96	340	C_22_H_44_O_2_
26	Oleic acid amide	26.93	1.33	281	C_18_H_35_NO
27	Nonadecanamide	26.93	1.33	297	C_19_H_39_NO
28	Hexadecanoic acid	27.81	1.21	640	C_38_H_76_O_5_Si
29	2-Ethylbutyric acid	27.96	4.95	396	C_26_H_52_O_2_
30	Lathosterol	29.33	15.30	386	C_27_H_46_O
31	Stearic acid,2,3-bis(trimethylsiloxy)	29.53	2.66	502	C_27_H_58_O_4_Si_2_
32	Trans-squalene	30.40	4.84	410	C_30_H_50_
33	Stigmastanol	30.80	5.29	562	C_32_H_51_F_5_O_2_
34	Cholestrane-3,16,26-triol	30.80	5.28	420	C_27_H_48_O_3_
35	Mandelic acid	31.08	1.42	380	C_20_H_36_O_3_Si_2_
36	Gamma-tocopherol	32.22	1.07	416	C_28_H_48_O_2_
37	22-Tricosenoic acid	32.37	2.23	352	C_23_H_44_O_2_
38	Vitamin E	32.91	2.59	430	C_29_H_50_O_2_
39	Thunbergol	33.71	1.59	290	C_20_H_34_O
40	Campesterol	35.09	12.85	400	C_28_H_48_O
41	Oleic acid	35.68	0.96	282	C_18_H_34_O_2_
42	Lauric acid, chloride	40.07	1.58	218	C_12_H_23_ClO

**Table 4:** Determination of acute toxicity (LD_50_) using Karber method.

Samples	Dose (mg/kg/bw)	Dose difference	No. of dead animals	Mean mortality	Dose difference × mean mortality	% Mortality
Control (distilled water)	0	0	0	0	0	0

*P. amygdalas* oil	100	0	0	0	0	0
	1000	900	0	0	0	0
	2000	1000	1	0.5	500	6.66

*J. regia* oil	100	0	0	0	0	0
	1000	900	0	0	0	0
	2000	1000	2	1	1000	13.33

Oil mixture	100	0	0	0	0	0
	1000	900	0	0	0	0
	2000	1000	1	0.5	500	6.66

*Note:* (*n* = 5). LD_50_ = highest dose − Σ (mean mortality × dose difference)/n. For *P. amygdalas* oil, LD_50_ = 2000 − (0 + 0 + 500)/5 = 1900 mg/kg/BW. For *J. regia* oil, LD_50_ = 2000 − (0 + 0 + 1000)/5 = 1800 mg/kg/BW. For oil mixture, and LD_50_ = 2000 − (0 + 0 + 500)/5 = 1900 mg/kg/BW.

**Table 5:** Effect of oil mixture on the number of pups/litters of *F*_1_ and *F*_2_ generation rats.

Parameter	*F*_1_				*F*_2_			
	Control	*T*_1_ (2 mL/kg)	*T*_2_ (4 mL/kg)	*p* value	Control	*T*_1_ (2 mL/kg)	*T*_2_ (4 mL/kg)	*p* value
Rats	4.50 ± 0.56	5 ± 0.36	5.83 ± 0.87	*p* > 0.05	4.17 ± 0.30	5.83 ± 0.60	6.83 ± 0.70^∗^	*p* < 0.05

*Note: n* = 6, Mean ± SEM; *F*_1_ presents first-generation pups, while *F*_2_ presents second-generation pups; *T*_1_ shows a low-dose group while *T*_2_ shows a high-dose group.

^∗^ *p* < 0.05 significant.

^∗∗^ *p* < 0.01 highly significant as compared to control.

**Table 6:** Reproductive findings in female rat.

Parameters	*F*_0_ Parents/*F*_1_ pups			*F*_1_ Parents/*F*_2_ pups			*p* value
	Control	*T*_1_ (2 mL/kg)	*T*_2_ (4 mL/kg)	Control	*T*_1_ (2 mL/kg)	*T*_2_ (4 mL/kg)	
No. of pregnant rats	4	5	6	6	6	6	*p* > 0.05
Litter size	6.75	6	5.33	4.16	5.83^∗^	6.83^∗^	*p* < 0.05
Fertility index (%) of *F*_0_ female	66.6	83.3	100	100	100	100	*p* > 0.05
Live birth index (%)	100	100	100	100	100	100	*p* > 0.05
Survival index at day 4 (%)	100	100	100	100	100	100	*p* > 0.05
Survival index at day 21 (%)	100	100	100	100	100	100	*p* > 0.05

*Note:F*_0_ presents parent generation, while *F*_1_ presents first-generation, *F*_2_ presents second-generation, *T*_1_ shows low-dose group while *T*_2_ shows high-dose group. *n* = 6, Mean ± SEM.

^∗^ *p* < 0.05 significant.

^∗∗^ *p* < 0.01 highly significant as compared to control.

**Table 7:** Male fertility parameters.

Parameters	Control	Ethanol (2 g/kg/d)	*T*_1_ (Vit E-105 IU/d)	*T*_2_ (oil Mixture-4 mL/kg/d)	*p* value
Semen pH	7.28 ± 0.03	7.27 ± 0.04	7.34 ± 0.04	7.30 ± 0.21	*p* > 0.05
Semen volume (mL)	2.05 ± 0.01	1.21 ± 0.04	2.33 ± 0.03	2.49 ± 0.05^∗^	*p* < 0.05
Sperm count (× 10^6^ sperm/mL)	62.0 ± 1.22	32.1 ± 5.92	61.53 ± 6.79^∗^	89.4 ± 8.22^∗∗^	*p* < 0.01
Sperm viability (%)	60.0 ± 5.50	22.4 ± 2.74	67.5 ± 6.19^∗^	95.6 ± 6.94^∗∗^	*p* < 0.01
Sperm motility (%)	50.80 ± 3.20	30.21 ± 3.82	62.31 ± 5.26^∗^	82.22 ± 6.71^∗∗^	*p* < 0.01

*Note: n* = 6. Mean ± SEM.

^∗^ *p* < 0.05 significant.

^∗∗^ *p* < 0.01 highly significant as compared to control.

## Data Availability

The supporting data will be available on request from the corresponding author.
